# Meta-ethnography 25 years on: challenges and insights for synthesising a large number of qualitative studies

**DOI:** 10.1186/1471-2288-14-80

**Published:** 2014-06-21

**Authors:** Francine Toye, Kate Seers, Nick Allcock, Michelle Briggs, Eloise Carr, Karen Barker

**Affiliations:** 1 Nuffield Orthopaedic Centre, Oxford University Hospitals NHS Trust, Oxford, UK; 2 Royal College of Nursing Research Institute, Warwick Medical School, University of Warwick, Coventry, UK; 3 School of Health and Life Sciences, Glasgow Caledonian University, Cowcaddens Road, Glasgow, UK; 4 Institute of Health and Well Being, Leeds Metropolitan University, Leeds, UK; 5 Faculty of Nursing, University of Calgary, Alberta, Canada; 6 Biomedical Research Unit, Nuffield Department Orthopaedics, Rheumatology and Musculoskeletal Sciences, University of Oxford, Oxford OX3 7LD, UK

## Abstract

Studies that systematically search for and synthesise qualitative research are becoming more evident in health care, and they can make an important contribution to patient care. Our team was funded to complete a meta-ethnography of patients’ experience of chronic musculoskeletal pain. It has been 25 years since Noblit and Hare published their core text on meta-ethnography, and the current health research environment brings additional challenges to researchers aiming to synthesise qualitative research. Noblit and Hare propose seven stages of meta-ethnography which take the researcher from formulating a research idea to expressing the findings. These stages are not discrete but form part of an iterative research process. We aimed to build on the methods of Noblit and Hare and explore the challenges of including a large number of qualitative studies into a qualitative systematic review. These challenges hinge upon epistemological and practical issues to be considered alongside expectations about what determines high quality research. This paper describes our method and explores these challenges. Central to our method was the process of collaborative interpretation of concepts and the decision to exclude original material where we could not decipher a concept. We use excerpts from our research team’s reflexive statements to illustrate the development of our methods.

## Correspondence

We aimed to build on the methods of meta-ethnography and explore the challenges of including a large number of qualitative studies. Syntheses of qualitative research in healthcare bring together qualitative research findings in order to facilitate knowledge transfer for improved healthcare. Recent reviews suggest that the number of qualitative syntheses in health care is dramatically increasing. Researchers have used different rigorous methods to produce qualitative syntheses [1-5]. Meta-ethnography has been used to synthesise qualitative findings [6], and is the most widely used method of qualitative synthesis reported [4]. Noblit and Hare propose seven stages of meta-ethnography which take the researcher from formulating a research idea to expressing the findings. These stages are not discrete but form part of an iterative research process. However, it has been 25 years since Noblit and Hare published their core text on meta-ethnography, and the current health research environment brings additional challenges to researchers aiming to synthesise qualitative research. For example: an exponentially increasing number of research reports; the expectations of the prevailing research community; the high value attributed to scientific methodologies in producing knowledge; and a more recent focus on the importance of knowledge translation as integral to the research process. Our paper describes and reflects on meta-ethnography as one method of synthesis in the context of a changing research landscape.

In 2011, Campbell and colleagues published an HTA review of meta-ethnography as a method of qualitative synthesis. In this review they argued that meta-ethnography is more suited to synthesising a smaller (n = 40) number of studies. We aimed to explore the challenges of including a larger number of studies, and were funded by the NIHR to complete a meta-ethnography of patients’ experience of chronic musculoskeletal (MSK) pain [7]. This paper describes our method, explores the challenges of using meta-ethnography to synthesise a large body of qualitative knowledge and develops and extends the methods proposed by Noblit and Hare. There are different ways of approaching meta-ethnography and we agree with Campbell and colleagues that a rigid methodological approach is not necessarily useful [6]. Our paper describes an innovative approach to meta-ethnography that reflects the contemporary research landscape. Our innovations enabled us to produce a meta-ethnographic synthesis that included 77 studies [7]. We explore the challenges of synthesising qualitative research (epistemological, cultural, practical and resource-based) and reflect on our decisions in the context of the health care research environment.

**Figure 1:**
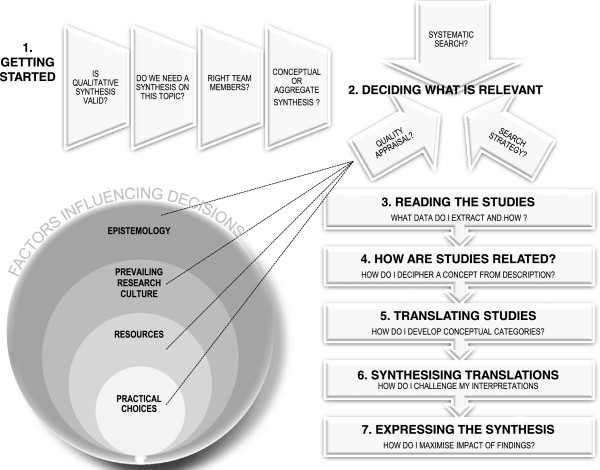
**Stages of Meta-ethnography, challenges and factors influencing decision-making.** Figure 1 illustrates the stages of Meta-ethnography, challenges and factors influencing decision-making. 1. Getting started; 2. Deciding what is relevant; 3. Reading the studies; 4. Determining how the studies are related; 5. Translating studies into each other; 6. Synthesising translations; 7. Expressing the synthesis.

Tong and colleagues propose the “ENTREQ” statement as a useful means of reporting the stages common to qualitative synthesis [8] and this is shown in Additional file 1. Figure 1 illustrates the seven stages of meta-ethnography: 1. Getting started; 2. Deciding what is relevant; 3. Reading the studies; 4. Determining how the studies are related; 5. Translating studies into each other; 6. Synthesising translations; 7. Expressing the synthesis. Figure 1 also illustrates specific challenges and factors that influenced our research decisions. Each team member wrote a reflexive statement at the beginning and end of the project, and we use excerpts from these statements as exemplars.

### Getting started

Noblit and Hare describe this stage of the research as ‘finding something that is worthy of the synthesis effort’ [9] (p 27). There are additional issues to consider at this stage: does research synthesis *fit* the qualitative approach; is a synthesis of this topic needed; what experience does the team need; what type of synthesis is appropriate; and what resources are available?

#### Does qualitative synthesis fit the qualitative approach?

A first consideration is whether or not we think that research synthesis fits a qualitative approach that focuses primarily on the *idiographic* or unique contextual experience, or whether synthesis removes us too far from the idiographic to reveal *truth?* In short, are the findings of qualitative *synthesis* valid? Whether we are synthesising a small or large number of studies, qualitative synthesis is still ‘an interpretation at least three times removed from the lives represented’ [1]; it is an interpretation of an interpretation of an interpretation. This problem is exacerbated by the issue of scale; the larger the number of studies that you include, the more difficult it is to maintain ‘sufficient familiarity’ with the original studies [6]. We agreed that qualitative synthesis is compatible with idiographic research if the interpretations remain firmly grounded in the primary qualitative studies. Our methodological innovations therefore hinged on developing methods to ensure that our interpretations remained grounded.

#### Is a synthesis of this topic needed?

The next consideration concerns the motivation for synthesising a particular topic. Noblit and Hare recommend a *keen interest* in the topic. We would argue that, in the contemporary landscape, a greater justification than ‘keen interest’ is necessary. Our decision to develop a conceptual synthesis of patients’ experience of chronic non-malignant MSK pain was sparked at the British Pain Society Annual General Meeting in 2009 when two of the research team (KS and FT) first met. We were aware of the large and growing body of qualitative research in this area (including our own). Our interest was shared by other clinicians and researchers at that meeting who expressed the need for a qualitative synthesis of chronic pain. The need for a knowledge synthesis could arise in various ways and settings; for example, to answer questions such as, why don’t patients take their medicine? [10]. Synthesising qualitative findings can make valuable knowledge accessible to healthcare professionals, particularly when the proliferation of studies might mean that this knowledge is ‘doomed never to be visited’ [1]. A preliminary search showed us that there was no qualitative synthesis specific to chronic musculoskeletal (MSK) pain, and confirmed that there was a growing body of qualitative knowledge in this area. Although existing syntheses do not preclude further ones, it is important to consider how any additional syntheses will build on existing knowledge.

#### What experience does the team need?

The next consideration is who contributes the necessary experience to the research team? Funders, rightly, demand that each team member makes a valuable contribution. In short *who* will bring *what* to the table? Our team included experienced health professionals and social scientists with ‘a keen interest’ in chronic pain and expertise specific to qualitative research synthesis. It could be argued that social science expertise is necessary to produce a ‘good’ synthesis, and our team did include social scientists. However, this raises the question of how we define a ‘social scientist’, or ‘expertise’.

I suppose it depends on your definition of social scientist . . . you do clearly need people who are prepared to see qualitative data as a valuable source of knowledge. You need to be able to think and reflect, and see parts and how they contribute to a whole. You need to be able to think conceptually. . . I think it depends much more on the individual, rather than the disciplinary label (reflexive statement).

These issues deserve thoughtful consideration. We agree that key skills are required; for example, a clear understanding and experience of qualitative analysis. These skills could be manifest in a range of people, including, but not exclusively, social scientists. Consider also the dynamics of the team; importantly, the team should provide the safety for each member to feel free to agree, disagree, or change their mind. Providing a learning environment which encourages individuals to express alternative, and even challenging, views can add rigour to qualitative research findings [11].

The group dynamics were clearly key and feeling “safe enough” to change your mind was important (reflexive statement).

Collaboration *‘requires that researchers be willing and able to risk voicing opinions not shared by everyone else in the group’*[12]*.*

We did listen to each other and challenge each other, which enhanced understanding and thus the review . . . a safe atmosphere to show one’s ignorance without fear of ridicule (reflexive statement).

It is important to consider who, beyond the researchers, would make a valuable contribution to the project; for example, patient and public representatives (PPI), clinicians and policy makers. Involving relevant stakeholders in the planning and execution of a qualitative synthesis helps to ensure that the knowledge is applicable and relevant, thus having a positive effect on knowledge translation. We set up an advisory group that included representatives from each of these groups. At times, we found it a challenge to engage patient representatives due to their other commitments and variable pain levels. If they could not attend a team meeting, we talked to them individually to ensure we included their perspective. Our research was based in an NHS hospital trust, and it was therefore possible to maintain the advisory input of clinicians. Decisions about team and advisory group membership, and how to communicate effectively, will be project specific and dependant on your aims and resources.

#### What type of qualitative synthesis is appropriate?

There are various methods for synthesising qualitative research [1-5]. For example, Barnett-Page and Thomas have identified: meta-narrative, critical interpretive synthesis, meta-study, meta-ethnography, grounded formal theory, thematic synthesis, textual narrative synthesis, framework synthesis and ecological triangulation [5]. The number of methodological approaches is likely to increase. A central distinction between synthesis approaches is (a) those that that aim to describe or ‘aggregate’ findings and (b) those that aim to interpret these findings and develop conceptual understandings or ‘theory’. As our aim was to develop conceptual understanding, rather than to aggregate findings, we agreed that meta-ethnography was an appropriate method of synthesis [9]. Consider which approach suits your research aim. Some authors argue that conceptual synthesis is more suited to a small number of studies; for example, Campbell and colleagues suggest around 40 studies are the maximum number to allow ‘sufficient familiarity’ [6]. We argue that conceptual syntheses of a large number of studies are both possible and useful. As description itself demands interpretation, it might be more useful to see aggregative and interpretive approaches as two poles on a continuum rather than two distinct approaches. Thus, irrespective of the size of your synthesis, consider where your approach falls along this continuum and which approach suits your research question. For example: Do you want to catalogue the qualitative themes arising (e.g. for the purposes of an outcome measurement questionnaire), or do you want a conceptual model that incorporates themes into a line of argument? (e.g. to increase your understanding of a particular experience or social process). This is important as researchers might not always consider which synthesis approach suits the specific research question.

#### What resources are available?

A final consideration to getting started is pragmatic; what resources (time, people, funding) are available? For example, studies may range from small scale projects aimed to inform clinical practice at a local level, to funded projects with a practice and policy focus. This will influence your decisions as each stage. For instance, do I have the resources to conduct a systematic review; is there money available for an experienced research team? What are my timescales? We were funded by the National Institute of Health Research (NIHR) to include a team experienced in qualitative research, specifically qualitative systematic reviews. Our team also included a senior research librarian. This resource is not always available and pragmatic decisions may be necessary (up to a point). Importantly, a larger body of existing knowledge will need a larger team of researchers. An important consideration for research stakeholders is the impact of available resources (or lack of) on the integrity of knowledge synthesis, and where, how and who to draw these lines.

### Deciding what is relevant

The next stage involves deciding what to include in your synthesis.

#### What is scope of search?

Defining the specific scope of the search is an important step in any systematic review. Your chosen area of study will influence the search strategy. For example, if very little is published about the topic, you may need to cast your search net more widely. Due to the sheer volume of studies exploring chronic pain, we defined very specific inclusion criteria and excluded a large body of research that did not meet our scope. For example, if the study did not allow us to disentangle the patient experience from that of others (e.g. carers, clinicians, and partners) then it was excluded. One of the challenges that we encountered was the absence of clear descriptions of study samples in the published abstracts. For example, we might not know the type of pain. This meant that we had to retrieve the full text of over 300 studies. A clearer description of the study sample in abstracts would facilitate more cost-effective and relevant qualitative research syntheses.

#### Do I need to do an exhaustive literature search?

Health research is proliferating and we have access to a vast and growing body of research. Researchers (and their funders) should consider whether or not an exhaustive search of the literature is necessary for qualitative syntheses. It could be argued that a disproportionate amount of time is spent searching for potential qualitative studies, and this time could have been better spent. However, researchers need to consider the prevailing research culture. We wanted to produce a conceptual analysis with a weight of evidence that would have resonance with the health research community who were more used to quantitative systematic reviews, and therefore chose to undertake a systematic search of the published literature. The Cochrane Collaboration has a major role in providing systematic reviews of high quality research. Part of their approach is a systematic search for all the evidence on a topic. Since expecting such a search is part of how high quality reviews are judged and used in practice, we felt it was important to follow this approach to ensure our findings were not dismissed as lacking rigour. A systematic search also gave us the unique opportunity to identify the qualitative studies published within our own area of interest and identify any gaps in knowledge, and to explore the usefulness of meta-ethnography for larger syntheses.

However, you could argue that systematic searching is not integral to high quality meta-ethnography. In their original text on meta-ethnography, Noblit and Hare do not advocate an exhaustive literature search, and the meta-ethnographies included in their core text include only 2–6 studies [9]. Reviews of published qualitative syntheses show that the number of studies included in meta-ethnographies ranges widely [2,4,6]. Meta-ethnography does not aim to summarise the entire body of available knowledge, or make statistical inference. Meta-ethnography focuses on conceptual insight, and including too many studies might make conceptual analysis ‘unwieldy’ or make it difficult to maintain insight or ‘sufficient familiarity’ [6]. Whereas in quantitative meta-analysis, omission of a key paper can have a dramatic effect on statistically drawn conclusions, some would argue that this is not true of conceptual qualitative synthesis which aims to develop ideas. Consider Campbell and colleagues’ view that ‘omission of some papers is unlikely to have a dramatic effect on the results’ [6]. Just as there is no consensus regarding the number of interviews necessary for a ‘good’ qualitative study [13], there is no consensus about whether or not you need to search for, and include, all available studies for a ‘good’ qualitative synthesis. This does not imply that meta-ethnography is not a rigorous research methodology. Importantly, there is a fundamental difference between qualitative and quantitative analysis that affects the decision of whether, or not, to search for and include all available studies. Namely, in qualitative research, analysis does not begin when all data is collected: Analysis and data collection occur simultaneously, often to the point where no new ideas are developing. Therefore, it may be that sampling strategies compatible with qualitative methodologies are more appropriate to qualitative synthesis. For example, you could stop searching for new data when ‘theoretical saturation’ is reached i.e. when collecting additional data seems to add no more insight [3,14]. However, the concept of data or theoretical ‘saturation’ could have its limitations; importantly, how do we know that an additional study will not add important insight?

I am worried about not having all papers. “Would there be that one paper which had a new insight” is always in the back of my mind. This is probably my quantitative systematic review training, but also the feeling that a particularly insightful author could come up with something new. I support the “data saturation approach” and think if the next twenty papers don’t offer anything new, what’s the likelihood of the twenty-first (reflexive statement).

Perhaps the answer to ‘how many’ is that we cannot know, and that ‘it depends’ [13]. If we can accept that there is a degree of uncertainty, what becomes important is that we include enough ‘data’ to ensure that our conceptual categories are robust, yet at the same time that the project remains grounded in the idiographic.

Did we need to include 77 studies? Would it have worked with 10, 20, 40 . . . .? Can we say any more on what would be ‘enough’? Maybe we can’t. Maybe this is where you need experienced qualitative researcher? That’s more questions than answers. Maybe this isn’t sortable and we have to live with ‘it depends’. Whilst this is true, it is also a bit of a cop out (Reflexive statement).

I suppose the skill in conceptual qualitative analysis is to make a decision whether or not you have enough ‘data’ to support a robust conceptual category. If you don’t then your analysis represents a catalogue or ‘aggregate’ of findings which may provide a lead for further sampling, but it is not necessarily conceptual (Reflexive statement).

#### Searching and screening

Another challenge specific to qualitative research is how to identify papers without being overwhelmed by the sheer number of hits. Strategies for identifying qualitative search can be unwieldy, and require ‘trade-offs’ between recall and precision [15]. In an evaluation of search strategies for qualitative research synthesis, Shaw and colleagues found that 96% of the initial search yield was not relevant. This means that search strategies for qualitative research can be over inclusive, time-consuming and expensive [15]. Accurate indexing and more explicit research designs in qualitative abstracts would facilitate more efficient searching. Our study supports the suggestion that screening for qualitative research syntheses will remain daunting [15]. The initial search yield of 24,992 studies was title-screened by two team members. If they were uncertain whether or not to include, they next screened the abstract, followed by the full paper. If after reading the full study they were still uncertain two other team members read the paper and made a final decision.

There are some useful resources for qualitative search filters. For example the InterTASC Information Specialists’ Sub-Group (ISSG) Search Filter Resource is a group of information professionals supporting research groups producing technology assessments for NICE [16]. From there you can access empirically-tested search filters for qualitative studies [17-20]. Shaw and colleagues also provide useful search filters and discuss their relative usefulness for qualitative syntheses [15]. We searched six electronic bibliographic databases (Medline, Embase, Cinahl, Psychinfo, Amed and HMIC) using the ISSG search filter resources. We did not use the *clinical query limits* option for qualitative research, as we found that this limit filtered out relevant qualitative studies. Consider also whether or not you intend to supplement the database search with other strategies. Hand searching specific journals is recognised as important for comprehensively identifying all relevant qualitative studies [15,19,21]. We identified specific journals that we knew agreed reported relevant qualitative research studies in full. These journals were: Journal of Advanced Nursing, Social Science and Medicine, Qualitative Heath Research, Sociology of Health and Illness and Arthritis Care and Research. Your own choices might differ depending on your topic. We subsequently added three journals that contributed the highest number of potential hits on the database searches. We further supplemented the search with citation checks. We did not search the grey literature and PhDs, partly due to the sheer volume of hits, and also because we aimed only to include peer reviewed and published reports. Decisions regarding search strategy and screening hinge upon your aims, resources, availability of studies and epistemological viewpoint. Importantly, do you think that a systematic search that aims to include every available study necessarily leads to more insightful knowledge? Our search strategy took six months of a two year study and 95% of the included studies were identified from three databases.

#### Quality appraisal

Although the use of quality criteria for qualitative research is debated, a growing number of researchers are choosing to appraise studies for qualitative systematic review. Hannes and colleagues report that the percentage of qualitative syntheses using quality appraisal increased from 40% (1988–2004) to 72% (2005–2008) [4]. However, although there are many frameworks suggested for appraising quality, there is no consensus about what makes a study good [6,22]. The decision to appraise, or not, is confounded by the prevailing research culture where gold standard methodologies are the expectation.

I might not do a quality appraisal if we did it again but still feel constrained by what the health community would think, so would probably feel I had to do it to get our findings used (reflexive statement)

We aimed to explore the issue of quality appraisal for qualitative synthesis [11] and used three methods of appraisal as a focus for discussion: First, the questions developed by the Critical Appraisal Skills Programme for appraising qualitative research (CASP) which has been used for appraising the quality of studies for meta-ethnography [10,23,24]. We assigned a numerical score to each question to indicate whether we felt that the CASP question had [1] not been addressed, [2] been addressed partially or [3] had been extensively addressed, thus giving a possible score range of 10–30. The CASP was useful in framing our discussions and encouraging us to read ‘carefully and systematically’ [6]. Secondly, the Qualitative Assessment and Review Instrument (JBI-QARI) designed by the Joanna Briggs Institute for use in systematic reviews of evidence [25]. Early in the appraisal process, we agreed the JBI-QARI did not add anything further than CASP to the final decision on inclusion. Finally, we categorised papers as either: a ‘key paper’ (‘conceptually rich and could potentially make an important contribution to the synthesis’); a satisfactory paper; a paper that is irrelevant to the synthesis; a methodologically fatally flawed paper [22]. This method has been used to determine inclusion of studies into meta-ethnography [26]. The concepts fatally flawed (FF) satisfactory (SAT) and key papers (KP) have not been defined, but are intuitive judgements made by a particular appraiser that comprise unspecified factors. Two team members appraised papers, and if they were unable to reach an agreement, the paper was sent to two other team members to make a final decision.

Our research supports the finding that where tools are used to appraise the quality of qualitative research, there is low inter-rather agreement [22]. It was striking that although ‘fatally flawed’ papers consistently scored below 20 on CASP, we found it extremely difficult to decipher, or agree about, what made a paper ‘key’ as opposed to a ‘satisfactory’ one [11]. This may illustrate that appraisal tools focus on methodological rather than conceptual strength. A common approach in quantitative research synthesis, recently adopted in qualitative synthesis, is to use sensitivity analysis to allow the reviewer to assess the impact of including ‘lower quality’ studies on the interpretation. For example, Carroll and colleagues used sensitivity analysis to show the possible benefits of quality appraisal for qualitative research synthesis [27]. However, this remains a challenge for qualitative synthesis if we do not agree about what good quality is.

Appraisal is time consuming and researchers (and funders) should consider *why* we appraise qualitative research. For example, do we include methodologically weak studies if they are conceptual rich?

I am still uncomfortable including anything that doesn’t have at least a semblance of decent methods, even if conceptually rich (reflexive statement).

The process I found most difficult to develop a clear view on was the process of critical appraisal. I am still not sure of the value of this although I still feel that some sort of quality appraisal is important. . . I would I think still feel uncomfortable including studies that have significant methodological limitations, but feel it is difficult to make a judgment as to where the line should be drawn (reflexive statement).

Dixon Woods and colleagues exclude studies they judge to be ‘fatally flawed’, and give some guidelines for determining this [3]. Others argue that excluding studies on the basis of quality criteria may mean that insightful studies are excluded [6]. Campbell and colleagues include ‘classic’ studies in their meta-ethnography, *assuming* ‘methodological integrity’ in the absence of fully reported methods. We felt that although conceptual richness was fundamental to meta-ethnography, the reported methods should be *good enough*, and agreed several factors were integral to methodological quality [11]. Importantly, does the study present a reflexive account of the research process that allows the reader to make a sound judgment about the authors’ interpretation? If we agreed that it did not do this, we did not include it in the synthesis.

### Reading the studies

This stage of meta-ethnography involves reading and re-reading the studies, in order to identify and describe the concepts. This requires ‘extensive attention to the details’ [9] (p28). This is not a discrete phase and thorough reading continues throughout. An important consideration at this stage is deciding *what* data to extract and *how* to do this. The raw data of meta-ethnography are ideas or concepts, which can appear in both the results and discussion sections. We wanted to be able to go back and re-read the original findings throughout so that we could compare developing ideas to the concepts as they were originally written. We did not use a data extraction form but rather uploaded a PDF version of the complete study onto NVivo 9 software [28]. This allowed us to code conceptual findings wherever they appeared within the paper, and compare individual team interpretations in one database. If the team member preferred to work from a paper version, FT transferred their memos onto the Nvivo database. NVivo is particularly useful for collaborative analysis as it allows you to keep a record and compare team member interpretations. NVivo 9 also allows the researchers to write and link memos to specific data in order to keep track of developing ideas. This software allowed us to classify certain study characteristics such as: author; journal; year of publication; type of pain; number and age of participants; source and country of participants (e.g. pain clinic in UK); method of data collection (e.g. interviews); methodological approach (e.g. grounded theory). In this way, we did not need to develop a data extraction form, and were able to go back and read the original paper many times over in order to remain grounded in the primary studies. FT also maintained an excel database of study demographics, appraisal scores and decisions on inclusion or exclusion.

### Determining how studies are related to each other

The purpose of careful reading in meta-ethnography is to identify and describe the ‘metaphors’ or concepts in studies and ‘translate’ or compare them to those in other studies. This is fundamental to meta-ethnography because concepts are the raw data of the synthesis. Determining how studies are related to each other involves creating ‘a list of key metaphors, phrases, ideas and/or concepts’ [9] (p 28). However, although Meta-ethnography requires clearly articulated concepts, it can sometimes be difficult to decipher these concepts through the description; to see ‘the wood through the trees’. For example, the reader may find themselves attempting to recode findings or to condense them into higher conceptual categories to make sense of them. One of the aims of qualitative analysis is to develop concepts that help us to understand an experience, rather than just describe that experience [29]. We describe a concept as a meaningful *idea* that develops by comparing particular instances. However, as the act of description itself requires a level of interpretation, it may be more useful to understand *description* and *concept* as two poles on a spectrum. Campbell and colleagues recognise this difficulty and did not distinguish findings from concepts [6].

Schütz’ concept of first and second order constructs [30] is frequently used in meta-ethnography studies, and is useful for distinguishing the data of meta-ethnography. Schütz makes a distinction between [1]*first-order constructs* (the participants’ ‘common sense’ interpretations in their own words) and [2]*second order constructs* (the researchers’ interpretations based on first order constructs). The ‘data’ of Meta-ethnography are second order constructs. In meta-ethnography, these second order constructs are then further abstracted to develop *third order constructs* (the researchers’ interpretations of the original authors’ interpretations). However, the distinction between first and second order constructs is not always straightforward as participants narratives are chosen by the author as exemplars of their second-order interpretation. Our approach deviates from other meta-ethnographies in that we based our synthesis entirely on clearly articulated second-order constructs. We did not *re-organise* or recode findings, but excluded data from analysis if we could not decipher a concept. We made this decision because of the methodological issues surrounding the re-organisation of data from qualitative research. The second order interpretation exemplified by narrative is based on a body of knowledge accessed through fieldwork. Therefore, attempts to re-organise findings without access to this wider body of knowledge might not illuminate the conceptual interpretation originally intended.

#### A collaborative approach to interpreting second order constructs

**Figure 2:**
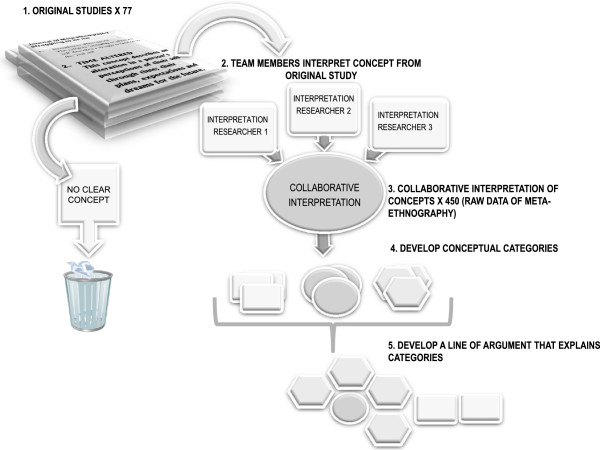
**Analysis.** Figure 2 illustrates the process of analysis from [1] 77 original studies, [2] team members’ interpretation of the concepts from the original studies, [3] developing collaborative interpretations of 450 concepts (the raw data), [4] developing conceptual categories through constant comparison and [5] developing a line of argument to explain the conceptual categories.

A fundamental issue with deciphering second-order constructs is that readers interpret concepts in light of their own experience. Thus different readers may suggest different interpretations. Thus a *meaningful idea* for one researcher may be only description for another. The reader makes a personal judgment about whether there is a relevant concept, and how to describe it. The unique methodological variance of our approach was to take a collaborative approach to interpreting second order constructs, in order to challenge our individual interpretations. In this way we were confident that our interpretations remained grounded in the original studies. In short, the interpretation of all 450 concepts entering the analysis was negotiated and constructed collaboratively. Figure 2 illustrates the process of collaborative interpretation of concepts and organisation into conceptual categories.

To do this, three members of the team read each paper to identify and describe their interpretation of each construct. The team then discussed and developed a collaborative interpretation of each concept. Due to the scale of the study and the number of concepts, our interpretations needed to combine clarity and precision in as few words as possible. We therefore used a combination of the author’s description of the second order construct (where it briefly and clearly described the construct), and our interpretation of the original construct (if the original was unclear or lengthy). In some cases, the primary author’s narrative exemplar was used as the most efficient concept descriptor. Our collaborative interpretations form the raw data of our synthesis, in the same way that interview narrative forms the ‘data’ of qualitative analysis. This approach allowed us to compile an inventory of concise interpretations of second order constructs that we felt confident were grounded in the primary studies [7]. The collaborative interpretation did not value one team member’s interpretation above another’s, but aimed to challenge individual interpretations and ensure that the final interpretation remained grounded in the original study.

#### Untranslatable concepts

If team members agreed that there was no clear concept articulated in the original source material, then we labelled it ‘untranslatable’ and did not include it in the analysis. For example, in some cases the construct consisted of a descriptive account or list of items that we felt the urge to ‘recode’. In other words, there was no central idea pulling the description together. This did not mean that the study was rejected in its entirety; some studies combined clearly defined and ‘untranslatable’ concepts. If one team member deciphered a concept, we included it in the analysis, even if another member did not. In this way, the untranslatable concepts identified became an exclusion criteria. Our aim was to challenge our interpretations, rather than reach consensus. Although this process was labour-intensive, we wanted to be confident that the concepts were grounded in the original studies. The three individual interpretations and resulting collaborative interpretations were entered onto NVivo 9. This allowed us to easily access the original study whilst reading the attached memos and developing ideas.

### Translating studies into each other

The next stage in meta-ethnography involves exploring how the second order constructs are related to each other and sorting concepts into conceptual categories or ‘piles’, thus ‘translating qualitative studies into one another’ [9]. ‘Translation’ is achieved through the constant comparative method [14]. Through constantly comparing constructs we begin to see similarities and differences between concepts and metaphors and organise them into further abstracted conceptual categories. In other meta-ethnographies, for example Campbell and colleagues [6], researchers have used an ‘index’ paper as a way of ‘orienting the synthesis’ [31]. In these examples, concepts from an early or ‘index’ paper are used to compare with concepts from subsequent studies. The decision to use an index paper may rest partly on the number of studies to be synthesised. We knew that this meta-ethnography would include a large number of studies, and comparing concepts across studies from an index paper in this way was likely to be unwieldy. There are also methodological issues to be considered if using an index paper to orientate analysis. One could argue that using an index paper is comparable to being constrained by a priori concepts. There is also the problem of how to decide which paper to use as an index paper, particularly as it can potentially have a dramatic effect on the resulting interpretation. Also, how do we define a ‘classic’ paper when there is no consensus about what makes a study ‘good’ [6,22,32]. We also need to consider that qualitative analysis does not start when the fully body of data is collected but continues alongside data collection. Thus we may not find the conceptually ‘richest’ study at the outset.

To translate studies into each other, all team members organised the body of concepts, through constant comparison, into categories or ‘piles’ which shared meaning. Each team member wrote a description for each category or ‘pile’. This process of categorisation using constant comparison is integral to qualitative research. The team met to discuss their categories and definitions. We did not aim to reach consensus, but to collaboratively develop our interpretations. At team meetings, members broke into separate groups and then re-grouped to discuss findings. Conceptual categories were written up on a white-board and discussed. Although team members gave different labels to their categories, there was an encouraging overlap in the individual category definitions. If we found second order constructs that did not ‘fit’ our developing conceptual categories, we went back to the original studies to challenge our interpretation and discussed the construct within the group. We also went back to the original studies after the final model was developed to check for fit.

**Figure 3:**
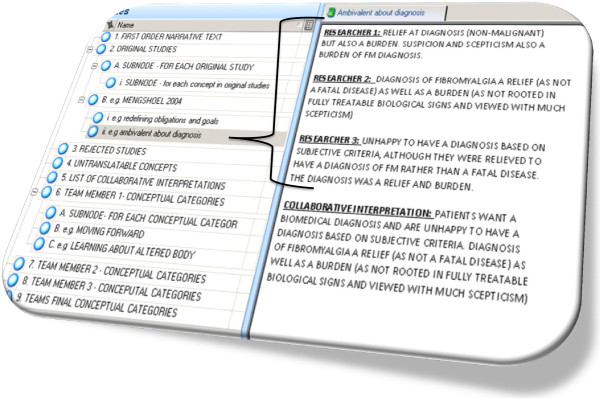
**Using Nvivo to organise analysis.** Figure 3 illustrates the nodes and sub-nodes used on NVivo 9 to organise the data extraction and analysis. It illustrates the process of creating collaborative translations from three interpretations in an attached NVivo 9 memo. The concept ‘Ambivalence about diagnosis’ is used as an example to show how a collaborative interpretation becomes part of the conceptual raw data for the meta-ethnography. The process of using NVivo 9 is described further in Additional file 2.

We combined the benefits of face-to-face team discussions with the benefits of using NVivo 9. Not all qualitative researchers would choose to use computer software to organise their data extraction and analysis. This is a matter of personal preference and we do not advocate a *right* way of doing it. Some researchers prefer to use a more ‘hands-on’ approach with pen, paper and scissors. We felt that this would be unwieldy with such a large number of studies. The principal investigator (FT) maintained and organised the NVivo 9 database. After each team meeting FT transferred the coding, categorising and supporting definitions and notes for each team member onto NVivo 9. This allowed her to compare how each team member had categorised and defined conceptual categories, whilst being able to return to the original article. Figure 3 illustrates how we used NVivo to organise data extraction and analysis, and this process is more fully described in Additional file 2 for those using NVivo software.

#### A worked example from second order to third order constructs

We describe a worked example of the process from an original concept to a conceptual category. Smith and Osborn describe the concept *‘negative impact on self’*[33]. They use exemplars from the patient’s own words (first order construct), for example:

It’s not who I am it’s just who I am if you know what I mean, it’s not really me, I get like that and I know like, you’re being mean now but I can’t help it. It’s the pain, it’s me, but it is me, me doing it but not me do you understand what I’m saying? (first order construct).

Smith and Osborn describe their concept in depth throughout the results (720 words), discussion (147 words) and conclusion (56 words) of the original paper, for example:

The notion of the self emerged in this study as an important aspect of the participants’ experience of chronic pain sensation, distress and disability. Their chronic pain assaulted and undermined their sense of self and the struggle to maintain a valued or coherent self was, at times, more unpleasant than enduring the physical sensation of pain (second order construct).

Three of our research team read Smith and Osborn’s paper and wrote our own interpretation of this concept:

Researcher 1: Pain can have a drastic effect on sense of self and identity; the ‘mean me’ as a result of pain outside self; engaged in battle against new self to keep ‘true self’; this struggle is more distressing than the pain itself

Researcher 2: Pain can impact one’s identity and perception of self significantly, resulting in denigrative mental inner conflict between the ‘two selves’: the ‘mean me’ and the ‘nice me’. There is a battle to ‘retain a good self’ and this struggle can be more distressing than even the pain itself.

Researcher 3: Negative impact of pain – pain influences behaviour so not the person really are – the nice and nasty (pain driven) parts of me.

FT then combined these interpretations into a concise collaborative interpretation that would become part of the body of data for the meta-ethnography.

*Smith and Osborn*[33]*: Negative impact on self*

Pain significantly impacts on one’s identity and perception of self, resulting in denigrative mental inner conflict between the ‘two selves’: the ‘mean me’ and the ‘nice me’. There is a battle to ‘retain a good self’ and this struggle can be more distressing than even the pain itself (collaborative interpretation).

All team members then sorted this concept into a ‘pile’ of other similar concepts which shared a common meaning. Each wrote a brief description of what this conceptual ‘pile’ described. Through discussion and comparison, we agreed that although we might use different labels for our ‘piles’ (e.g.: striving to be normal me; body and self in conflict; impact on self; changed self; still me and not me) our contents and descriptions overlapped sufficiently to be incorporated into a conceptual category that we named ‘struggling to affirm a sense of self’. Through constant comparison, we repeated the same process for all 450 concepts identified. Full details of the concepts and conceptual categories are available elsewhere [7].

### Synthesising translations

Once we had developed conceptual categories or ‘piles’ and concisely described each pile, the next stage of meta-ethnography is to synthesise or make sense of these categories. This may differs from other synthesis approaches that stop analysis at the stage where they have theoretically saturated categories. *Synthesising Translations* is an on-going process where findings are further abstracted to form a conceptual framework. Although Noblit and Hare distinguish seven stages of meta-ethnography, these stages are not discrete. They suggest three ways of synthesising translation for meta-ethnography; [1]*refutational* syntheses (where findings contradict each other), [2]*reciprocal* syntheses (where findings are directly comparable); [3] findings are taken together and interpreted as a *line of argument.* We intended to develop a line of argument synthesis, which involves ‘making a whole into something more than the parts alone imply’ [9] (page 28). This is achieved by constantly comparing concepts and developing ‘a grounded theory that puts the similarities and differences between studies into interpretive order’ [9] (page 64).

Drawing on team discussions, and using NVivo 9 to continually compare original studies, concepts, conceptual categories and team memos, we collaboratively developed a visual structure of categories that made sense of the developing analysis. Each team member considered whether or not the developing structure reflected the discussions that had taken place. If a team member did not think that a particular concept or category fitted the line of argument, we discussed this in meetings and made necessary changes. We constructed a diagram to develop and refine our line of argument [7]. This diagram was developed collaboratively over time and was the main focus of team discussions during this phase. Several amended versions of this diagram were created until we arrived at a model that expressed our final team interpretation.

The *findings* of qualitative research will inevitably be only one possible interpretation of data. Different team members bring ideas and points of view into the analysis. The interpretative nature of qualitative research challenges the prevailing scientific research culture which aims to reduce, or even remove, the effect of researcher bias.

I worry that an interpretation of an interpretation will be dismissed by more quantitatively orientated colleagues. Although I feel confident our processes are rigorous, the change in understanding required from others who see reducing “bias” as essential is a challenge. (Reflexive statement).

Although we regard interpretation as a strength of a conceptual research model, we propose that *challenging* our interpretations is integral to qualitative research rigour [11]; in other words rather than removing bias, we challenge personal interpretation through collaboration. We made great efforts to work collaboratively to question our individual interpretations at each stage. This facilitated a dialectic process where our ideas were challenged and modified. Meta-ethnography is an interpretive form of knowledge synthesis which aims to develop new conceptual understandings. This process is iterative and utilises an on-going form of knowledge production (thesis-antithesis-synthesis). Therefore bringing ideas into a study is not a limitation, as long as a priori ideas are challenged. In this way, Blumer distinguishes between *definitive* concepts that precisely define the object of enquiry, and sensitizing concept, which give ‘a general sense of reference and guidance in approaching empirical instances’ [34] (page 7).

### Expressing the synthesis

This phase concerns the dissemination of the research findings to maximise their impact. In line with a recent focus on optimising knowledge translation, effective dissemination and impact is a critical component of all research. However, the success of knowledge translation from research is complex. An expectation of health research culture is to produce peer-reviewed publications, and to a degree, our expertise is evaluated by what is published. However, the proliferation of research publication increases the danger that findings are not reaching the right target audience.

The key things are getting it to a meaningful audience where it has potential to change practice and do justice to the patients’ voice versus chasing a journal with a good citation index (reflexive statement).

Publishing and conference papers feed our own research and academic agendas but can seem so futile in the wall of ignorance facing people with chronic pain (reflexive statement).

Involving relevant stakeholders from the beginning can facilitate effective and appropriate knowledge transfer. It may also be useful to consider other means of dissemination alongside more conventional methods (peer-reviewed publications, presentations, teaching, conferences). Active measures to promote knowledge transfer (KT), ‘the exchange, synthesis and application of research results’ [35] (page 1), should be seriously considered. Chalmers and Glasziou suggest that as much as 80% of money invested in research is wasted, partly through ineffective KT [36]. However, despite increasing investment and the requirement to demonstrate the impact of research, the research-practice gap remains [36-38]. One of our planned outputs from the meta-ethnography was a short film, ‘Struggling to be me’, produced in collaboration with a media agency based at Bournemouth University (Red Balloon). This film, produced from a script constructed from narrative interviews and performed by an actress, is available on NIHR Youtube [39]. The film received around 3, 500 hits in the first six months. Performative social science [40] uses non-traditional media, such as drama or film, to perform research findings and maximise knowledge translation [41]. In the process of presenting research findings through film, the focus shifts to whether these findings evoke, provoke and stimulate ideas [42]. Our monthly team meeting included ‘Impact Plan’ as a regular agenda item. On-going impact activities include: research in collaboration with Cardiff University where the film has been utilised as part of a teaching module on pain; collaboration with Pain Concern UK [43]; a feature in the Hot Topics GP Update course for GPs [44]; a contribution to the patient voice in the Royal College of General Practitioners guidelines for engagement with commissioners [45]. One of the issues to consider within the impact plan is ensuring that the time allocated to impact is adequately funded. In short, impact is on-going and is unlikely to fit neatly into a window on a Gantt chart. More research to explore the utility of innovative methods for maximising the impact of qualitative research would useful.

### Conclusions factors influencing research decisions

This paper describes an innovative approach to meta-ethnography that not only reflects the contemporary research landscape, but also allowed us to produce a large meta-ethnographic synthesis that included 77 original studies. Other researchers have used different rigorous methods to produce conceptual syntheses. Reviews of published qualitative syntheses show that only a few meta-ethnographic syntheses include more than 40 studies [2,4,6]. There are also other synthesis approaches that include a larger number of studies, or that combine qualitative and quantitative reports [5]. Some of the challenges that we discuss are exacerbated by the scale, for example ‘deciding what is relevant’; other challenges are present irrespective of scale, for example, do we use an index paper or not? The innovation of our study was to develop a method of meta-ethnography that allowed us to produce a conceptual synthesis grounded in a large number of original studies. The process through which we developed collaborative interpretations, and through which we excluded original material if we could not decipher a concept, was integral to our innovative method.

The challenges of qualitative synthesis hinge upon epistemological and practical issues that need to be considered alongside the prevailing health research communities expectations about what determines high quality research. The factors influencing our decisions were multifactorial. For example, the decision to quality appraise, or not, is influenced by several factors: pragmatic (how shall I do it?), resource-based (how much time and how many people do I have available?), epistemological (can this method improve our knowledge?) and cultural (is this method considered valid by the research community?). Pragmatic and resource-based challenges include: do we need a synthesis; do I have the right team; what is my search strategy; how do I extract and manage data; how do I decipher concepts from findings; how do I challenge my interpretations?

We had to balance striving to prove rigour in our processes with making the system manageable and deliverable within the project resources. (Reflexive statement)

Epistemology concerns *what* truth, or knowledge, is and *how* we meaningfully acquire it. For example, if we think that knowledge is constructed within a specific historical and social context, is it possible (or desirable) to strip away the context to reveal an objective truth? These challenges are integral to health research which seeks *true* or *valid* findings on which to base excellent clinical practice. A useful review of the epistemological challenges inherent to qualitative methods can be found in a Health Technology Assessment (HTA) report by Murphy and Dingwall [32]. Epistemological challenges pertinent to meta-ethnography include: can qualitative synthesis reflect *true* experience; is it necessary to include all studies from a systematic search; is quantity integral to the quality of synthesis; is quality appraisal compatible with qualitative synthesis, and if so, how do we judge conceptual richness? Health research takes place within, and is constrained by the prevailing scientific research community where systematic review, quality appraisal and objectivity is an expectation of rigour. At the same time, those attempting to synthesise qualitative research can equally find themselves constrained by the expectations of the qualitative research community; for example, analysing *too many* interviews or studies might be interpreted as unwieldy and as leading to superficial analysis. Qualitative researchers in health care who synthesise qualitative health research can thus find themselves caught between a rock (medical research culture) and a hard place (social science research culture). Research communities would benefit from exploring their similarities, and meeting the challenges of ‘uncertainty and contingency’ in collaboration with each other [46] (page 884).

Our processes which strengthened our rigour were just as robust and just as flawed as [quantitative systematic reviews] (reflexive statement).

Like a quantitative systematic review, there are many decisions to be made in the process. Perhaps quantitative reviews don’t always acknowledge this. (reflexive statement)

Our suggested method for conducting large meta-ethnographies develops Noblit and Hare’s seminal work and makes and important contribution to the methodology of qualitative systematic review. Ultimately, the aim of qualitative research syntheses in healthcare is to contribute to improvements in clinical care and patient experience. By increasing our knowledge of patient experience through qualitative enquiry, we can contribute to improvements in care. However, in order to have an impact on healthcare practice, the research must be considered good enough and then be accessible. A final consideration is what measures we take to translate the knowledge from qualitative findings into practice. More research is needed to explore the impact of qualitative research on relevant stakeholders and how we maximise the impact of qualitative research in order to improve care. High Quality research synthesis should not end with the final write up.

## Competing interests

This project was funded by the NIHR Health Services and Delivery Research programme (project number 09/2001/09).

The views and opinions expressed therein are those of the authors and do not necessarily reflect those of the NIHR HS&DR programme, NIHR, NHS or the Department of Health.

## Authors’ contributions

FT and KS originated the idea for the meta-ethnography of chronic MSK pain on which this manuscript it based. All authors contributed to the development of the method, and read and approved the final manuscript. FT drafted the first version of the manuscript. FT, KS and KB made a significant contribution to the ideas developed and presented in this manuscript.

## Authors’ information

FT is a fellow of the Royal Anthropological Institute with a master’s degree in Archaeology and Anthropology. She is also a qualified physiotherapist with an interest in chronic pain management. She has expertise and interest in qualitative health research and methodology.

KS has a quantitative and qualitative health and pain research background and expertise, and has used mixed methods in most of her research. Her professional background is nursing.

NA is a doctoral qualified nurse academic and practising pain nurse.

MB has broad experience in systematic reviews. She has completed syntheses of qualitative research using Joanna Briggs QARI methodology. Her professional background is nursing.

EC qualified as nurse and throughout her twenty five year research career has utilised mixed methods in her pain research.

KB is a qualified physiotherapist, with experience of running chronic pain management programmes.

## Pre-publication history

The pre-publication history for this paper can be accessed here:

http://www.biomedcentral.com/1471-2288/14/80/prepub

## Supplementary Material

Additional file 1 Shows a statement for enhancing the transparency in reporting the synthesis of qualitative research (ENTREQ) as proposed by Tong and colleagues (8). Click here for file

Additional file 2 **This appendix provides the coding structure that we used to organise data extraction and analysis.** The appendix is intended for those who are familiar with using Nvivo for coding qualitative data. Click here for file
