# Supplementary material for: Meta-ethnography 25 years on: challenges and insights for synthesising a large number of qualitative studies
Source: BMC Med Res Methodol. 2014 Jun 21;14:80. doi: 10.1186/1471-2288-14-80 (PMC4127190; doi:10.1186/1471-2288-14-80)
Supplement: Additional file 1 — Shows a statement for enhancing the transparency in reporting the synthesis of qualitative research (ENTREQ) as proposed by Tong and colleagues (8). [file 1471-2288-14-80-S1.docx]

**APPENDIX 1: ENTREQ statement(**[**5**](#_ENREF_5)**)**

| Aim | To utilise existing research knowledge to improve understanding and thus best practice in patient care for chronic non-malignant MSK pain. |
| --- | --- |
| Synthesis Methodology | Meta-ethnography - to develop conceptual interpretation of included studies. |
| Approach to searching | Comprehensive search strategy to seek all available studies. |
| Inclusion criteria | Full published reports of qualitative studies that explored adults’ experience of chronic non-malignant musculoskeletal pain. Chronic was defined as lasting for 3 months or more. Limited to English Language |
| Data Sources | Six electronic databases up until February 2012 (Medline, Embase, Cinahl, Psychinfo, Amed and HMIC) supplemented by hand-searching contents list of specific journals for 2001-2011 and citation tracking. |
| Electronic Search strategy | We combined terms available from the InterTASC Information Specialists’ Sub-Group (ISSG) Search Filter Resource (16) withsubject headings and thesaurus terms to identify relevant studies. |
| Study Screening methods | A research librarian and researcher screened the titles of the identified articles. If they were uncertain whether to include the study after reading the title, then FT read the abstract. If she was still uncertain about inclusion, the full text was checked by another researcher. If they remained uncertain the article was sent to the full team for a consensus decision. |
| Study Characteristics | See tables 4 and 5 in Toye and Colleagues 2013 ([7](#_ENREF_4)) |
| Study selection results | See figure 4 in Toye and Colleagues 2013 ([7](#_ENREF_4)) |
| Rationale for appraisal | We agreed that conceptual insight was fundamental to meta-ethnography, but also felt that papers should be reported ‘well enough’ methodologically to allow us to make a judgement about the inductiveness of the findings. We felt that we needed some assurances that the report was inductive and grounded in patients’ experience. |
| Appraisal items | 1. Critical Appraisal Skills Programme for appraising qualitative research (CASP). Available at <http://media.wix.com/ugd/dded87_951541699e9edc71ce66c9bac4734c69.pdf> (accessed today) 2. [Qualitative Assessment and Review Instrument (JBI-QARI)](https://legacyowa.warwick.ac.uk/Exchange/assgav/Inbox/article%20on%20quality.EML/1_multipart_xF8FF_2_appraisal%20article%2022nov11.doc/C58EA28C-18C0-4a97-9AF2-036E93DDAFB3/Qualitative%20Assessment%20and%20Review%20Instrument%20(JBI-QARI)%20designed%20by%20the%20Joanna%20Briggs%20Institute%20(http:/www.joannabriggs.edu.au/Appraise)%20to%20manage,%20appraise,%20extract%20and%20synthesize%20qualitative%20data%20as%20part%20of%20a%20systematic%20review%20of%20evidence) ([25](#_ENREF_24)) 3. Categorised papers as either: a ‘key paper’ (‘conceptually rich and could potentially make an important contribution to the synthesis’); a satisfactory paper; a paper that is irrelevant to the synthesis; a methodologically fatally flawed paper ([22](#_ENREF_12)). |
| Appraisal process | Two team members (FT and JA) appraised all papers. If they did not agree, the paper was sent to two other team members to make a final decision. |
| Appraisal results | See Table 1 in Toye and Colleagues 2013 ([7](#_ENREF_4)) |
| Data extraction | PDF version of the complete study loaded onto NVivo 9 software for analysing qualitative data ([28](#_ENREF_28)). Data extracted from results and discussion sections |
| Software | Nvivo 9 |
| Number of reviewers | Seven |
| coding | Three team members read each paper to identify and describe their interpretation of each concept within the primary studies. The team discussed and developed a collaborative interpretation of each concept. |
| Study comparison | ‘translating qualitative studies into one another’ ([9](#_ENREF_6))is achieved through the constant comparative method ([14](#_ENREF_14)). |
| Derivation of themes | Line of argument synthesis or ‘interpretive order’ developed through constant comparison and collaboration. |
| quotations | The data of meta-ethnography are the concepts derived from each included study. We use primary narrative as exemplars of these concepts. See Table 7 in Toye and Colleagues 2013 ([7](#_ENREF_4)). |
| Synthesis output | Figures 6 and 7 in Toye and Colleagues 2013 ([7](#_ENREF_4)) |
