# Supplementary material for: Meta-ethnography 25 years on: challenges and insights for synthesising a large number of qualitative studies
Source: BMC Med Res Methodol. 2014 Jun 21;14:80. doi: 10.1186/1471-2288-14-80 (PMC4127190; doi:10.1186/1471-2288-14-80)
Supplement: Additional file 2 — This appendix provides the coding structure that we used to organise data extraction and analysis. The appendix is intended for those who are familiar with using Nvivo for coding qualitative data. [file 1471-2288-14-80-S2.docx]

# Additional file2 Coding for meta-ethnography using Nvivo

This appendix provides the coding structure that we used to organise data extraction and analysis. The appendix is intended for those who are familiar with using Nvivo for coding qualitative data. The aim is to provide a helpful structure for organising large meta-ethnography studies. Specific functions are not given in detail as they will vary between versions of Nvivo. There is no right or wrong way to organise qualitative data and others may have different preferences. However, central to coding for large meta-ethnographies is being able to keep a track of, and challenge, the coding and conceptual development whilst being able to return to the original studies. A particularly useful function during analysis is the Nvivo ‘ VIEW CODING STRIPES’ which allows you to observe multiple levels of coding related to a section of data.

We used the following node and sub-node structure as illustrated in figure 3:

- NODE: FIRST ORDER NARRATIVE TEXT
- NODE: ORIGINAL STUDIES
  - *SUBNODES*: ORIGINAL STUDIES
    - Concepts from original studies
      (memo attached describing researchers’ interpretation of each concept)
- NODE: REJECTED STUDIES
- NODE: UNTRANSLATABLE CONCEPTS
- NODE: LIST OF COLLABORATIVE INTERPRETATIONS
  (collects togetherall collaborative interpretations from memos)
- NODE: TEAM MEMBER 1 – CONCEPTUAL CATEGORIES
  - *SUBNODES*: FOR EACH CONCEPTUAL CATEGORY
    (memo attached describing team member’s conceptual category)
- NODE: TEAM MEMBER 2 ETC. – CONCEPTUAL CATEGORIES
  - *SUBNODES*: FOR EACH CONCEPTUAL CATEGORY
- NODE: TEAM FINAL CONCEPTUAL CATEGORIES
  - *SUBNODES*: FOR EACH CONCEPTUAL CATEGORY
    (memo attached describing team’s final conceptual category)

# Nvivo Method

## DATA EXTRACTION

1. Import external PDF or word copy of each original study into Nvivo database.
2. As you read each paper, code all first order narrative under a separate node (FIRST ORDER NARRATIVE TEXT). This will allow you to identify narrative sections if you chose to use this material.
3. Set up a Node for REJECTED STUDIES. This will allow you and the team to review rejected papers later.
4. Set up a node (ORIGINAL STUDIES) and a sub-node for each original study. Code the entire paper into that sub-node. This will allow you to track which study concepts come from when you view coding stripes.
5. Under each ORIGINAL STUDY sub-node, set up further sub-nodes for each identifiable concept in that paper. Code the section of the paper that refers to each concept into its uniquesub- node. In most studies, these concepts are identified by sub-headings, although in some cases concepts might not be clearly marked. It is useful to use the concept name given by the primary author so that you can compare your interpretation to the original source material at any stage.

You will now have a series of sub-nodes listed under the original studieswhich include the original source material for the concepts to be included in the meta-ethnography. The next stage is to create a list of collaborative interpretations of this source material.

## COLLABORATIVE INTERPRETATION OF CONCEPTS

Each researcher reads the study and writes a description of their interpretation of each concept in it.

1. Attach a MEMO to each concept sub-node. This memo is to include each researcher’s description of the concept along with the collaborative interpretation based on these individual descriptions. This COLLABORATIVE INTERPRETATION OF CONCEPTS becomes the raw data of the meta ethnography
2. Set up a node ‘COLLABORATIVE INTERPRETATION OF CONCEPTS and code all the collaborative interpretations from the memos under this node. This will give you a list of collaborative interpretations that are the raw data of the analysis. These interpretations are grounded in the original text and are based on a reflexive collaborative process.
3. Set up a node for ‘UNTRANSLATABLE CONCEPTS’ to include any original material that the team agree cannot be defined as a concept. This will allow the team to review this decision later and refer back to source material.

## ANALYSIS

1. Set up a unique node for each team member where each researcher can create their own series of sub-nodes to organise the data. By setting up a unique node for each researcher, you will be able to track individual coding and thus challenge the developing conceptual analysis. In short you will be able to observe how each researcher has coded the concepts.
2. Set up a node for the final TEAM CONCEPTUAL CATEGORIES. This will be used to re-organise the researcher’sunique sub nodes into a structure that makes sense of the individual categories. As each author has their own unique coding node you will still be able to track how each individual has coded. This process of comparing coding challenges the interpretations at each stage and adds rigor to the analysis.
3. Attacha memo to each conceptual category node toincludethe individual and teamdescriptions of the developing CONCEPTUAL CATEGORIES. This allows you to keep track of how the category is developed over time.

## LINE OF ARGUMENT

At this point you have a structure of nodes and memos which describe the conceptual categories to be included in a line of argument.
